# Serum short chain fatty acids mediate hippocampal BDNF and correlate with decreasing neuroinflammation following high pectin fiber diet in mice

**DOI:** 10.3389/fnins.2023.1134080

**Published:** 2023-04-11

**Authors:** Jamie S. Church, Jane A. M. Bannish, Leighelle A. Adrian, Kimberly Rojas Martinez, Asari Henshaw, Jared J. Schwartzer

**Affiliations:** Program in Neuroscience and Behavior, Department of Psychology and Education, Mount Holyoke College, South Hadley, MA, United States

**Keywords:** pectin fiber, diet, SCFAs, BDNF, hippocampus, cytokines

## Abstract

**Introduction:**

Dietary components, such as prebiotic fiber, are known to impact brain chemistry *via* the gut-brain axis. In particular, short chain fatty acids (SCFAs) generated from excessive soluble fiber consumption are thought to impact neuroimmune signaling and brain function through increased production of neurotropic factors. Given reports that high dietary fiber intake is associated with increased mental health and improved quality of life scores, we set out to identify whether changes in SCFA levels as a result of a high soluble fiber diet mediate hippocampal neuroinflammation and brain derived neurotrophic factor (BDNF) in mice.

**Methods:**

Adult male and female C57BL/6 mice were fed a 1-month high pectin fiber or cellulose-based control diet. Following 1 month of excessive pectin consumption, serum SCFAs were measured using gas chromatography–mass spectrometry (GC-MS) and hippocampal cytokines and BDNF were assessed *via* multiplex magnetic bead immunoassay.

**Results:**

Pectin-based fiber diet increased circulating acetic acid in both sexes, with no effect on propionic or butyric acid. In the hippocampus, a high fiber diet decreased TNFa, IL-1ß, IL-6, and IFNγ and increased BDNF levels. Furthermore, increased SCFA levels were inversely correlated with neuroinflammation in the hippocampus, with acetic acid revealed as a strong mediator of increased BDNF production.

**Conclusion:**

Collectively, these findings highlight the beneficial effects of fiber-induced molecular changes in a brain region known to influence mood- and cognition-related behaviors. Dietary composition should be considered when developing mental health management plans for men and women with an emphasis on increasing soluble fiber intake.

## 1. Introduction

Dietary composition represents an important environmental factor that impacts the gut microbiome and subsequent communication across the gut-brain-axis. For example, high fiber diets (HFD) rich in soluble prebiotic carbohydrates are purported to improve brain function by altering gut health (Silva et al., 2020; Guan et al., 2021). Excessive consumption of prebiotic fiber supports growth of probiotic intestinal microbes that ferment dietary fiber and release short chain fatty acids (SCFAs) into circulation (Holscher, 2017). SCFAs enhance production of hippocampal-supporting growth factors, such as brain derived neurotrophic factor (BDNF), and influence mood- and cognition-related behaviors (Wei et al., 2014; van de Wouw et al., 2018). A soluble fiber-deficient diet in rodents results in loss of biodiversity within the microbiome, increasing gut permeability, systemic inflammation, and impaired brain function (Shi et al., 2021). While there is clear evidence that soluble fiber provides health benefits, not enough is known about the biological mechanisms linking SCFA availability to the underlying brain health improvements reported from HFD.

Exposure to dietary fiber increases SCFAs and neuroinflammatory changes in brain areas known to influence mood and cognition including the hippocampus. Acetate, propionate, and butyrate metabolites are commonly generated following HFD consumption, and changes in these SCFAs can be detected in the cecum (Burokas et al., 2017; Matt et al., 2018; Szklany et al., 2020) feces (Romo-Araiza et al., 2018) and serum (Shi et al., 2021). Noticeable changes in these metabolites begin by 1 month of HFD in males, however, it remains unknown whether a similar timeline exists in females. This is important given the recent prospective study in women identifying the benefits to a diet high in soluble fiber on mental health and quality of life scores (Ramin et al., 2020). Importantly, rodent studies have noted key impacts of HFD on brain function through altered neuroinflammation (Matt et al., 2018; Paderin and Popov, 2018; Romo-Araiza et al., 2018) and changes in growth factors, most notably BDNF, and these studies predominantly focus on the use of only male rodents (Vazquez et al., 2015; Burokas et al., 2017; Szklany et al., 2020). While these studies support a link between dietary fiber and systemic signaling changes across the gut-brain axis, they are limited in their inclusion of female mice and do not identify a mechanistic link connecting HFD, subsequent serum SCFA signaling, and brain inflammation in both sexes.

Short chain fatty acids are important for the structure and neuroimmune function of the brain’s immune cell, microglia. Of note, acetate, propionate, and butyrate aid in the normal development of brain microglia, and deficits in microglial maturity and formation in germ-free mice can be restored with a 4-week administration of these SCFAs in drinking water (Erny et al., 2015). Acetate in particular was shown to reduce lipopolysaccharide- (LPS) induced microglial reactivity and IL-1β expression in rat models of neuroinflammation (Reisenauer et al., 2011; Soliman et al., 2012). Similarly, *in vitro* administration of butyrate induces microglial process elongation toward an anti-inflammatory ramified morphology (Wang et al., 2018) and epigenetically reduces expression of pro-inflammatory genes (Patnala et al., 2017). *In vivo* models of neuroinflammation also demonstrate the beneficial anti-inflammatory effects of butyrate on brain health. For example, treatment with sodium butyrate can be neuroprotective against ischemic stroke through epigenetic regulation of inflammatory genes in microglia (Patnala et al., 2017). Moreover, butyrate-induced epigenetic regulation in the hippocampus decreases depressive behavior following LPS-induced microglial reactivity (Yamawaki et al., 2018). These demonstrated anti-inflammatory effects of SCFAs on microglial function suggest that increasing SCFA availability through consumption of a HFD can have beneficial effects on brain health.

While previous research has documented behavioral and molecular effects of prolonged HFD, we sought to investigate how serum concentrations of SCFAs mediate the effects of HFD on neuro-immune cytokine expression and BDNF concentrations in the hippocampus, a brain region known to influence mood and cognition. A better understanding of how HFD influences brain signaling in females and males will provide new insights into the link between dietary fiber and brain health. We hypothesized that SCFAs generated from consumption of a high pectin fiber diet support beneficial neuroinflammatory signaling mechanisms regardless of sex. Adult male and female C57BL/6 mice were fed a high pectin fiber or control diet for 1 month and then serum concentrations of acetic, propionic, and butyric acids (SCFAs) were measured using GC-MS. In addition, hippocampal protein concentrations of TNFα, IL-1β, IL-6, IFNγ, and BDNF were measured using magnetic bead-based immunoassay, a rapid and highly sensitive multiplexing approach to analyte quantification (Vignali, 2000; Kellar et al., 2001), and analyze concentrations were correlated with serum SCFAs using causal mediation analysis.

## 2. Materials and methods

### 2.1. Animals

Forty-seven 8-week-old C57BL/6 mice [23 female (F), 24 male (M)] were obtained from 6 litters within a colony maintained at Mount Holyoke College originally sourced from breeding pairs purchased from Jackson Laboratory (Bar Harbor, MA, USA). Mice were maintained in a temperature-controlled (23°C) vivarium on a 12 h light/dark cycle (lights on at 08:00 h) and housed in individually vented cages of 2–5 same-sex littermates. Prior to the start of the study, mice were raised from birth on a standard 2018 Teklad global 18% protein rodent diet containing 14.7% neutral detergent fiber, which is an estimate of insoluble fiber, including cellulose, hemicellulose, and lignin (Envigo, Madison, WI, USA). Food and water were provided *ad libitum*. All procedures were approved by the Institutional Animal Care and Use Committee (IACUC) at Mount Holyoke College and in accordance with the guidelines provided by the National Institutes of Health Guide for the Care and Use of Laboratory Animals.

### 2.2. Diet manipulation

Dietary manipulation began at 8 weeks of age and was maintained for 1 month. All mice from within a single cage were randomly designated to the high soluble fiber group (pectin-based) (13F/11M) or control diet (10F/13M) group containing insoluble cellulose-based fiber. The diets used were: Teklad Custom Diet #: TD.94045 (Envigo, Madison, WI, USA); AIN-93G Purified Diet (control) and Teklad Custom Diet #: TD.170554 (Envigo, Madison, WI, USA); 10% Pectin Diet (93 G, VI, G) (high fiber) (Table 1). Diet was provided *ad libitum* and weighed weekly, beginning at the onset of exposure, to assess consumption. In addition, mice were weighed weekly to measure growth and general health throughout the study.

**TABLE 1:** Dietary components.

	Control	High fiber
(g/KG)	TD.94045	TD.170554
Casein	200	200
L-cystine	3	3
Corn starch	397.486	342.134
Maltodextrin	132	132
Sucrose	100	100
Soybean oil	70	70
Cellulose	50	–
Pectin	–	100
Mineral mix, (94046)	35	35
Vitamin mix, (94047)	10	15
Choline bitartrate	2.5	2.75
Vitamin K1, phylloquinone	–	0.002
TBHQ, antioxidant	0.014	0.014
Green food color	–	0.1
% by weight		
Protein	17.7	17.7
Carbohydrate	60.1	55.6
Fat	7.2	7.2
% kcal from		
Protein	18.8	19.8
Carbohydrate	63.9	62.1
Fat	17.2	18.1

TBHQ, tert-Butylhydroquinone.

### 2.3. Tissue collection

Following 32 days of diet, mice were anesthetized using 3% isoflurane gas. Cardiac blood was collected in a 1 mL syringe using a 26-gage needle, allowed to clot at room temperature (RT) for >40 min and centrifuged for 10 min at 10,000 × *g* at 4°C. Serum was stored at −80°C until preparation for GC-MS. A subset of anesthetized mice (8F/8M per diet) were decapitated and whole brains were collected, snap frozen in liquid nitrogen, and stored at −80°C.

### 2.4. Serum metabolites analysis with GC-MS

Short chain fatty acid extraction, derivatization, and quantification using GC-MS were conducted using a modified version of methods outlined in Zhang et al. (2019). Extraction procedures began with 50 mL of serum diluted 1:1 in d/dH_2_O and combined with 10 mL of 5M HCl. Next, 100 mL of anhydrous diethyl ether (DE) was added to each sample, vortexed, left on ice for 5 min, and centrifuged at 10,000 × *g* for 5 min. The DE layer (∼90 mL) was transferred to a new tube containing a few crystals of anhydrous Na_2_SO_4_. The remaining aqueous layer was further extracted two times with DE in the original tube and each extracted DE layer pooled together. For derivatization, 100 mL of the DE extracted sample was transferred to a glass insert, combined with 5 mL N,O-bis(trimethylsilyl)trifluoroacetamide (BSTFA), and vortexed before incubating at 37°C for 2 h and stored tightly capped at RT until GC-MS. Samples were analyzed by GC-MS using a Phenomenex ZB-5ms column (30 m × 0.25 mm × 0.25 μm) on an Agilent 6890 GC/5973 MSD (Agilent Technologies, Santa Clara, CA, USA) system, using helium as carrier gas at 1 mL/min. 1 μL of sample was injected in splitless mode with an injector temperature 260°C. Oven parameters were 40°C for 2 min, ramped at 15°C/min to 150°C with 1 min hold followed by 50°C/min to 300°C for a total run time of 15 min. Mass spectra were measured in SIM mode, monitoring ions at m/z 75, 117, 131, 145, and 159 with 50 ms dwell time. Three samples were lost, two from the female control and one from the male fiber groups.

### 2.5. Brain cytokine analysis

The hippocampus was dissected from frozen whole brains using a 2 mm coronal section from Bregma: −1 mm to Bregma: −3 mm. A 1.5 mm diameter biopsy punch was centered over each side of the hippocampus, positioned according to the coronal C57BL/6J mouse brain atlas (Rosen et al., 2000). Hippocampus punches were stored at −80°C until homogenization. Brains were collected from a random subset of mice from each sex and treatment condition (*n* = 8/group).

Bilateral hippocampus punches were homogenized in 3.85 μL buffer per mg tissue; buffer contained Factor 1, Factor 2, and 2 mM PMSF dissolved in Bio-Plex cell lysis kit (Bio-Rad, Hercules, CA, USA; catalog #:171304011). Homogenates were then mixed with buffer for 20 min at 4°C in an orbital shaker, centrifuged at 4°C at 4,500 *g* for 4 min, and the resulting supernatant was used for subsequent analyses. Supernatants of each sample were diluted 1:50 in cell lysis buffer and protein concentrations determined using the Pierce Bicinchoninic acid (BCA) Protein Assay Kit (ThermoFisher Scientific, Waltham, MA, USA; Cat #23225). A 5 mg/mL protein concentration of each supernatant was used in the subsequent cytokine analysis to adjust for total protein in all samples.

Cytokine analysis was carried out in duplicate using Luminex Multiplex Immunoassay (Mouse High Sensitivity T Cell Magnetic Bead Panel; Millipore Sigma, Burlington, MA, USA; Cat #:MHSTCMAG-70K) by research assistants blinded to diet group. TNFα, IL-1β, IL-6, IFNγ, IL-10 cytokines, and BDNF growth factor were measured in 1 mg/mL dilution of each sample. The minimum limit of detection for each cytokine was as follows: TNFα: 0.41 pg/mL; IL-1β: 2.58 pg/mL; IL-6: 0.54 pg/mL; IFNγ: 0.15 pg/mL; IL-10: 0.53 pg/mL; BDNF: 0.42 pg/mL. The diluted samples were combined with 25 μL premixed magnetic bead solution containing beads for each of the 5 cytokines measured and incubated overnight at 4°C in the dark. The following day, the plate was washed three times, 25 μL of detection antibody was added, and left to incubate at RT for 1 h in the dark. Then, 25 μL of Streptavidin-Phycoerythrin was added and left at RT to incubate for 30 min in the dark. The plate was then washed three times and 150 μL of drive fluid added before being stored overnight at 4°C in the dark. The next day the plate was shaken for 20 min at RT and read on a MAGPIX system (Luminex, Austin, TX, USA) using xPONENT 4.2 software. Unknown sample concentrations were determined from a standard of known concentrations (manufacturer provided) using a 5-parameter logistic regression curve. All cytokines with a bead count <50 were excluded.

### 2.6. Data analyses

Data were analyzed using R version 4.0. Food consumption was evaluated using a 3-way repeated-measures ANOVA with diet and sex as between-subjects factors and week as within-subjects factor. Given that mice were group housed with littermates, and the potential impact of litter-to-litter variations on microbiome, percent weight gain, serum SCFAs, and brain protein concentrations were evaluated using mixed-effects modeling to control for Type I error as a result of litter effects (Lazic and Essioux, 2013; Lazic et al., 2018). Model parameters were fit using the “nlme” package and constructed using forward stepwise regression. Model fit was evaluated using maximum likelihood ratios test and the best model was selected based on the Akaike information criteria (AIC). First, a basic random effects model was constructed with litter set as a random slope. Then, the fixed effects of diet and sex were added to the model and tested for model fit using the log likelihood ratio test compared to a random-effects only model. Finally, a full model included fixed main effects and a diet by sex interaction and tested against the model containing the fixed effects. The final model was assessed graphically to verify the data met the assumptions of linearity, normality, and homogeneity of variance. Cytokine values below the limit of detection were included in calculation as the value one half the limit of detection.

To identify highly associated relationships between brain cytokines, BDNF, and serum SCFA concentrations, bivariate correlations were examined using Pearson’s r correlation coefficient without adjustments. Mediation analyses were calculated for the effect of diet and SCFA on BDNF levels in the hippocampus. First, individual regression models were constructed for the exposure-mediator relation with diet used as the independent variable and SCFA concentration as the outcome measure. Then, a second model for the full effect was created with both diet and SCFA as predictors and BDNF levels as the outcome variable. Mediation was evaluated using the “mediation” package in R statistics software. Briefly, percentage mediated was calculated by dividing the indirect effect through the mediator by the total effect, calculated as the sum of coefficients from the indirect and direct effects. Mediations were run with 1,000 simulations using the quasi-Bayesian Monte Carlo method. All data are represented as mean and standard error of the mean (SEM). Differences between group means for all outcomes were considered significant if *p* < 0.05.

## 3. Results

### 3.1. Food intake was transiently reduced by HFD without affecting weight gain

**FIGURE 1:**
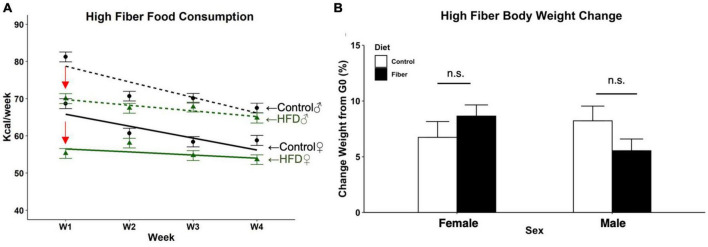
Food consumption and body weight change following 1 month of high fiber diet. **(A)** Estimated weekly food consumed per animal (Kcal/week); **(B)** percent change in body weight after 4 weeks of HFD. *N* = 3 cages/group, cage total Kcal divided by the number of mice per cage for food consumption data; *n* = 10–13/group for body weight. Data are represented as mean + SEM; repeated measures 2-way ANOVA. Female (solid line), male (dotted line), high fiber diet (green), control diet (black), significant diet by week interaction (red arrow). n.s. not significant.

Food consumption and weight gain were measured weekly to assess the metabolic effects of a 1-month soluble fiber diet. For food consumption, there were significant main effects for diet (*p <* 0.01), sex (*p* < 0.001), and week (*p* < 0.001) (Figure 1A). Significant interactions of diet and week (*p* < 0.0001) and sex and week (*p* < 0.001) were also observed. For the first week of diet exposure, both female and male mice fed HFD (females: 55.3 Kcal, 95% CI: 52.3–58.3; males: 70 Kcal, 95% CI: 66.9–73.1) consumed less than control chow-fed groups (females: 68.7 Kcal, 95% CI: 65.7–71.7, *p <* 0.001 vs. control chow; males: 81.2 Kcal, 95% CI: 78.2–84.2, *p <* 0.001 vs. control chow). By week 2 there were no significant differences between groups based on diet, which continued for the duration of the study with the exception in week 4 of HFD-fed female mice (53.6 Kcal, 95% CI: 50.6–56.6) consuming less food than sex-matched controls (58.8 Kcal, CI: 55.8–61.8).

Over the 1-month study, the percent change in body weight between the first and last day of diet in all mice revealed no effect of diet (*p* = 0.241) or sex (*p* = 0.811) on final weight gain (Figure 1B). There was a significant sex by diet interaction with female HFD mice having a significantly higher percent gain in weight compared to male HFD mice, *t*(38) = −2.188, *p* = 0.035. However, no differences were observed between HFD and control diet within either sex.

### 3.2. HFD increased SCFAs in both male and female mice

**FIGURE 2:**
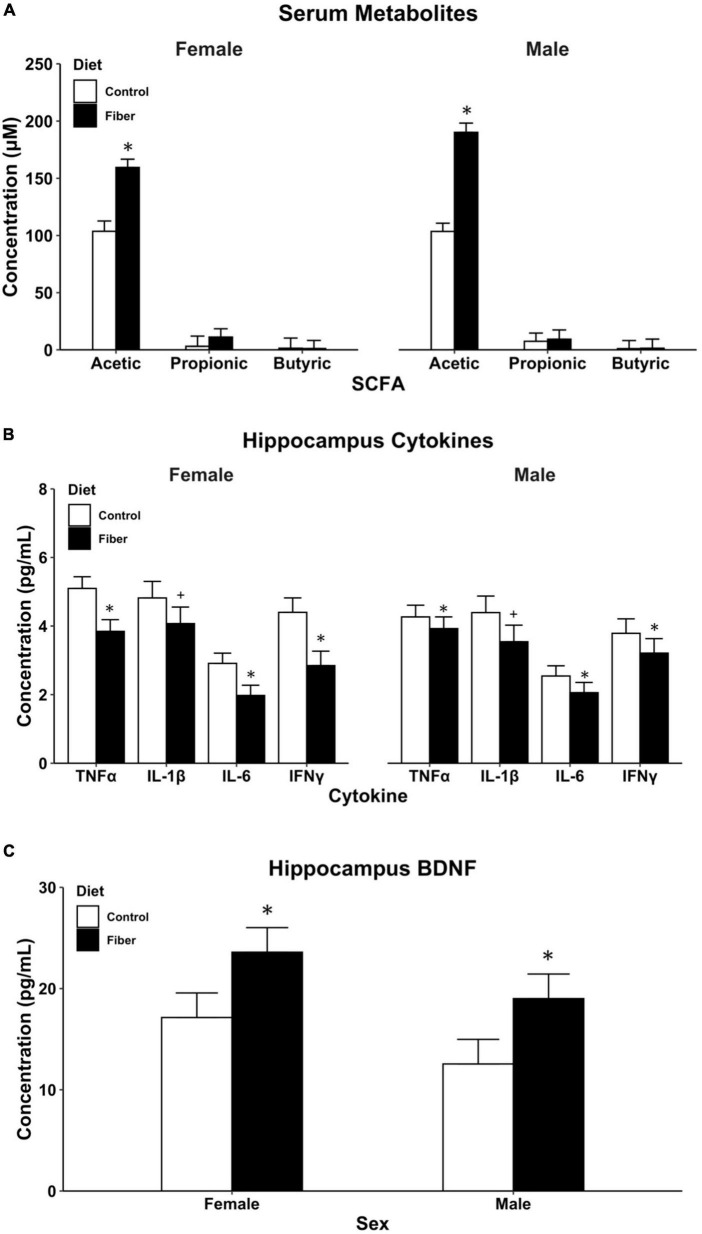
Biological measures following 1 month of HFD. **(A)** SCFA metabolites measured in serum using GC-MS; **(B)** cytokine levels in the hippocampus; and **(C)** BDNF levels in the hippocampus. Hippocampus proteins **(B,C)** measured using multiplex magnetic bead immunoassay. Data are represented as mean + SEM. *p < 0.05, ^+^*p* < 0.1 vs. control as determined by linear mixed effects modeling with diet and sex as fixed effects and litter as random effects. *n* = 8–13/group. Control diet (white), high fiber diet (black).

Following 1 month of HFD, serum was collected from male and female mice and analyzed for SCFA concentrations using GC-MS. To control for pseudo-replications due to within-cage and within-litter consanguinity, SCFA concentrations were estimated using forward step-wise mixed-effects modeling with litter set as a random slope (Supplementary Tables 1–3). For acetic acid, a model with fixed effects for sex and diet was chosen (*p* = 0.0029), and the addition of a diet by sex interaction term did not significantly improve model fit (*p* = 0.18). Male and female mice fed a pectin-based fiber diet had an average increase of 72.25 μM (95% CI: 32.38–112.12) concentration of acetic acid compared to mice fed a cellulose-containing control diet, *t*(4) = 4.86, *p* = 0.0083 (Figure 2A). No differences in acetic acid levels were detected between male and female mice, *t*(37) = 1.30, *p* = 0.20. For both propionic acid and butyric acid, models containing only the random-effect of litter were selected (Supplementary Tables 2, 3) given that the addition of diet and sex as fixed effects did not improve model fit for either serum SCFA (propionic, *p* = 0.41; butyric, *p* = 0.40). Therefore, concentrations of propionic and butyric acid were not significantly different between diet or sex groups.

### 3.3. HFD reduced hippocampal inflammation and increased BDNF

Model parameters were estimated using linear mixed-effect modeling with litter as a random intercept. For IFNγ, IL-1β, IL-6, and TNFα, the best fit model included fixed effects for both sex and diet with no interaction term (Supplementary Tables 4–7). A 1-month diet of soluble fiber (HFD) resulted in an average of 2.8 pg/mL (95% CI: 1.54–4.06) lower concentration of IFNγ in the hippocampus of both male and female mice compared to cellulose-fed control mice *t*(26) = −4.34, *p* < 0.001. No effect was observed for sex *t*(26) = 0.08, *p* = 0.94. Similar diet-induced reductions in hippocampal cytokines were observed in both sexes for IL-6, *t*(26) = −2.58, *p* = 0.016 and TNFα, *t*(26) = −3.11, *p* = 0.0045, and a trend toward significance in IL-1β *t*(26) = −1.73, *p* = 0.09 (Figure 2B). For the anti-inflammatory associated cytokine IL-10, concentrations were below the limit of detection in both pectin and cellulose-fed mice.

For BDNF measures, a mixed-effects model was chosen with sex and diet as fixed effects and litter as a random effect (Supplementary Table 8). The addition of an interaction term to the model had only a marginal improvement in model parameters that did not reach statistical significance (*p* = 0.082). Pectin-based HFD resulted in an average 6.5 pg/mL increase in BDNF concentration in the hippocampus compared to cellulose-containing control diet (95% CI: 0.97–11.94). These increases in BDNF concentration were statistically significant, *t*(26) = 2.30, *p* = 0.029, for male and female mice. No differences were observed in BDNF concentrations between sexes, *t*(26) = −1.63, *p* = 0.11 (Figure 2C). Taken together with the reduced cytokine expression, these findings demonstrate that excessive consumption of pectin fiber reduced hippocampal neuroinflammation and improved neurotrophic concentrations in both male and female mice.

### 3.4. Serum SCFAs are associated with neuroinflammation and BDNF in the hippocampus

**FIGURE 3:**
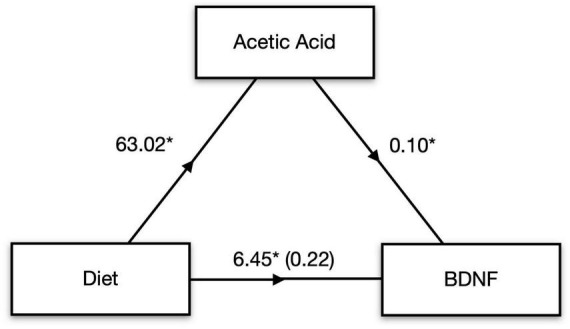
SCFAs mediate diet-induced changes in brain signaling. Mediation analysis revealed that serum acetic acid mediates the effect of high fiber diet on BDNF levels in the hippocampus. *p < 0.05.

Concentrations of all four detectable cytokines in the hippocampus were positively correlated with one another, indicating a strong association between these pro-inflammatory markers (Table 2). Moreover, these cytokine measures were negatively correlated with BDNF concentrations including IFNγ, *r* = −0.49, *t*(30) = −3.08, *p* = 0.004; IL-6, *r* = −0.42, *t*(30) = −2.54, *p* = 0.016; and TNFα, *r* = −0.37, *t*(30) = −2.16, *p* = 0.039. Interestingly, there was a moderately strong and statistically significant positive correlation between serum acetic acid levels and concentrations of BDNF in the hippocampus, *r* = 0.49, *t*(27) = 2.89, *p* = 0.007, suggesting a potential mechanistic link between pectin-based HFD and brain neurotrophic availability. To further explore this link between fiber, SCFAs, and subsequent BDNF concentration in the hippocampus, we performed a mediation analysis. Serum acetic acid concentration significantly mediated the link between dietary fiber and hippocampal BDNF concentration, average causal mediated effect (ACME) = 6.49, *p* < 0.001, 95% CI: 1.50–12.38, with the proportion of indirect to total effect (I/T) = 0.91 (Figure 3). In fact, the direct effect of diet on BDNF concentrations in the hippocampus was no longer significant after accounting for the role of serum acetic acid, average direct effect (ADE) = 0.22, *p* = 0.90, underscoring a potential causal role for SCFA levels in the serum in mediating the impact of HFD on brain health.

**TABLE 2:** Correlation matrix of hippocampal BDNF, cytokines, and serum SCFAs.

	BDNF	IFNγ	IL-1β	IL-6	TNFα	Acetic	Propionic
IFNγ	−0.49**	–					
IL-1β	−0.23	0.71***	–				
IL-6	−0.42*	0.80***	0.61***	–			
TNFα	−0.37*	0.82***	0.69***	0.82***	–		
Acetic	0.49**	−0.35	−0.14	−0.36	−0.28	–	
Propionic	0.31	−0.45*	−0.28	−0.33	−0.36	0.28	–
Butyric	0.26	−0.42*	−0.31	−0.38*	−0.32	0.08	0.64***

Bold values indicate statistically significant correlation. *p < 0.05, **p < 0.01, ***p < 0.001.

While no other SCFA correlated with BDNF concentration, we did observe several significant negative correlations between SCFAs and hippocampal cytokines. In particular, IFNγ was negatively correlated with both propionic acid, *r* = −0.45, *t*(27) = −2.60, *p* = 0.015, and butyric acid, *r* = −0.42, *t*(27) = −2.40, *p* = 0.023. Moreover, there was a marginally significant negative correlation observed between serum levels of butyric acid and IL-6 in the hippocampus, *r* = −0.38, *t*(27) = −2.14, *p* = 0.04. These negative correlations in the absence of a statistically significant effect between HFD and control groups suggests that basal concentrations of SCFAs in serum may be associated with brain inflammation even in the absence of a measurable effect of diet.

## 4. Discussion

High dietary fiber intake is associated with increased mental health and improved quality of life scores in both men and women (Fernstrand et al., 2017; Ramin et al., 2020), and growing evidence suggests that the type of fiber and influence on gut microbiome can impact the function and health of the central nervous system (for review see Foster and Neufeld, 2013). While observational studies and a recent meta-analysis have noted a link between dietary fiber and brain health (Saghafian et al., 2022), few preclinical studies have been carried out to identify a clear networking of the molecular markers along the gut-brain axis that underly these links. Importantly, the type of dietary fiber, soluble or insoluble, impacts the rate of fermentation by gut microbiota and differentially influences brain function. More specifically, a diet high in soluble fiber, as compared to insoluble fiber, has greater positive impacts on mental health and inflammation (Barber et al., 2020). Given the links between dietary fiber and brain health in both men and women, we set out to identify how the consumption of a high soluble fiber diet impacts brain inflammation and whether these changes are sex-dependent. Specifically, we hypothesized that SCFAs generated from consumption of a high pectin fiber diet support beneficial neuroinflammatory signaling mechanisms in both male and female mice. Our findings identified that increasing serum acetic acid mediated elevations in hippocampal BDNF and furthermore, higher level of BDNF correlated with concomitant decreases in markers of neuroinflammation. Moreover, changes in both butyric acid and propionic acid negatively correlated with IFNγ and IL-6 in the hippocampus. These findings further support the proposed link between the soluble fiber pectin and reduced brain inflammation, regardless of sex, through changes in circulating SCFAs.

Pectin is a polysaccharide soluble fiber often found in plant-based foods (Voragen et al., 2009). Both animal and human studies have demonstrated the protective effects of a high pectin diet on intestinal wall integrity through an immune-modulatory mechanism (Jiang et al., 2016; Vogt et al., 2016; Sun et al., 2017; Sahasrabudhe et al., 2018; Merheb et al., 2019; Wilms et al., 2019; Beukema et al., 2020). Moreover, HFD can be protective against peripheral immune-system challenges (e.g., endotoxin-induced sickness behavior) by shifting splenocytes and macrophages toward a T helper-2 mediated immune response. In the central nervous system, a 4-week exposure to a high soluble fiber diet in male Balb/c mice showed decreased gene expression of several pro-inflammatory cytokine in microglia (Matt et al., 2018). Similarly, we observed decreases in IFNγ, IL-1β, IL-6, and TNFα in the hippocampus of male and female C57Bl6/J mice fed a high pectin diet for 1 month, an observation that closely mirrors the down-regulation observed in Matt et al. (2018). Despite these similarities, the behavioral and immunological responses to dietary fiber are not always consistent (Merheb et al., 2019) and are thought to be dependent on microbiome composition and mouse strain. A recent study in C57Bl6/J mice demonstrated that the behavioral benefits of HFD vary based on cohort and subsequent microbiota composition (Mailing et al., 2019), a finding that could explain some of the challenges in reproducibility. While microbiome analyses were not conducted in the current study, our observed immunomodulatory effects of a high pectin diet in the hippocampus support the reported mental health and quality of life benefits observed in humans and underscore the need to identify specific mechanistic pathways linking dietary fiber to brain health (Fernstrand et al., 2017; Ramin et al., 2020).

While much of the research on brain cytokine signaling and the impact of diet on brain health has focused on models of brain dysfunction, it is important to also identify any biologically relevant effects of diet-induced changes to neuroinflammation in health individuals. Importantly, typical diets consumed in countries across Europe and North America lack sufficient levels of recommended fiber (Barber et al., 2020) and these nutritional deficiencies are closely linked to rates of mental health in adults (Wattick et al., 2018). Our study using healthy adult mice demonstrate that changes in dietary fiber can have measurable changes in cytokine concentrations in the absence of any predisposed psychiatric or degenerative disorder, an observation that underscores the importance of maintaining proper nutrient intake. While long-term deficiencies in fiber across the lifespan are known to impact mental health, it remains unknown whether the reductions in cytokine concentrations observed in our healthy adult mice have any long-term biologically relevance. Importantly, cytokines in the central nervous system play key roles in neurotransmitter signaling pathways by modulating synthesis, reuptake and metabolism of several neurotransmitters including serotonin, dopamine, and glutamate (Miller et al., 2013). Moreover, cytokines play essential roles in neuroplasticity under physiologically relevant conditions that impact neurogenesis, synaptic scaling and remodeling, long-term potentiation, and learning and memory (Stellwagen and Malenka, 2006; Yirmiya and Goshen, 2011). Given these links between cytokine signaling and neurocircuitry functions, our observed decreases in pro-inflammatory cytokines in the hippocampus of male and female mice suggest that improvements in dietary composition may be important for the maintenance of proper mental health in healthy individuals (Raison et al., 2006; Czirr and Wyss-Coray, 2012; Miller et al., 2013).

Dietary fiber is used by anaerobic bacteria as fermentation substrates, and SCFAs are common metabolites produced by fermentation that are thought to impact gut-immune function (Nicholson et al., 2012). Butyrate, acetate, and propionate are the three most abundant SCFAs produced from soluble fiber digestion and their availability and concentration is dependent on the type of fiber consumed as well as microbiota composition (Macfarlane and Macfarlane, 2012). For pectin, the major fiber source used in the composition of our HFD, acetic acid is produced in the highest proportion compared to butyrate and propionate (Smith et al., 1998). We observed a high concentration of serum acetic acid in mice from both pectin (HFD) and cellulose-based (control) diets, with a significantly higher concentration of acetic acid observed in the high soluble fiber pectin-based diet. While our analyses were limited to serum SCFAs, it is also possible that a more robust diet-induced increase could have been detected in the cecum or feces (Tian et al., 2016). Previous studies have also demonstrated increases in acetic acid following both pectin and inulin as the source of fiber (Matt et al., 2018; Singh et al., 2020), and the presence of these SCFAs have known central nervous system effects. Studies note the impacts of SCFAs in down-regulating pro-inflammatory cytokines (Cox et al., 2009) and increasing BDNF in the frontal cortex (Wei et al., 2014). Our findings extend these previously observed neurotrophic effects by identifying an increase in hippocampal BDNF in response to a HFD that is positively correlated with increasing acetic acid. Indeed, administration of acetate in mice was recently observed to promote brain plasticity and increase BDNF mRNA expression in the hippocampus (Marrocco et al., 2022) underscoring the potential mechanistic role for serum acetic acid elevations to enhance brain plasticity and function.

In addition to the link between levels of peripheral acetic acid concentration and hippocampal BDNF, levels of propionate and butyrate negatively correlated with pro-inflammatory cytokines in the brain. These findings are in line with previous work measuring fiber-induced increases in cecal acetate, propionate, and butyrate along with elevated BDNF in the prefrontal cortex of male mice following 11 weeks of dietary supplementation with fructo- and galacto-oligosaccharide beginning at birth (Szklany et al., 2020). Similarly, 10 weeks of these oligosaccharides were shown to increase BDNF in the hippocampus of adult male mice (Burokas et al., 2017), and even moderate 4–5 week exposure to soluble fiber can elevate cecal or fecal butyrate and acetate, increase BDNF, and reduce cytokines including TNFα and IL-1β in the hippocampus (Vazquez et al., 2015; Matt et al., 2018; Romo-Araiza et al., 2018). Conversely, 15 weeks of fiber deficiency in adult mice decreased serum acetate, propionate, and butyrate, increased gut permeability, and increased hippocampal TNFα, IL-1β, and IL-6 (Shi et al., 2021), further demonstrating the importance of SCFAs in regulating brain inflammation. In fact, our mediation analysis identified acetic acid as a substantial contributor linking the effects of dietary fiber to BDNF concentrations in the hippocampus. Specifically, 4 weeks of a pectin-based fiber diet in our study increased circulating SCFA concentrations in the serum and these increases, particularly in acetic acid, correlated with the increases in neurotrophic factors (BDNF). Together, the decreases in neuroinflammation and elevations in BDNF highlight an important neuro-modulatory effect of dietary fiber through gut-brain axis signaling.

## 5. Conclusion

In total, our findings indicate that moderate short term HFD rich in soluble pectin fiber increases serum acetic acid, reduces hippocampal neuroinflammation, and increases BDNF. Furthermore, these serum and brain changes are correlated with one another and are present in both male and female mice, which has important implications for modulating diet to influence brain health. This work is an important addition to the limited preclinical data available assessing the neurobiological effect of soluble fiber in females, and contributes a novel networking of the molecular markers along the gut-brain axis that underly the link between dietary fiber and brain health. Taken together with previous work, our findings demonstrate that beneficial HFD-induced molecular changes in serum are present by the 1-month time point and changes in neuroinflammation are independent of sex. Overall, dietary composition should be considered when developing mental health management plans for men and women, with an emphasis on increasing soluble fiber intake.

## Data availability statement

The raw data supporting the conclusions of this article will be made available by the authors, without undue reservation.

## Ethics statement

The animal study was reviewed and approved by the Mount Holyoke College Institutional Animal Care and Use Committee.

## Author contributions

JC: conceptualization and methodology. JS: formal analysis. JB, LA, KR, and AH: writing–review, editing, and investigation. JC and JS: writing–original draft, visualization, and supervision. JS, JC, KR, AH, and LA: funding acquisition. All authors contributed to the article and approved the submitted version.

## References

[B1] Barber T. M. Kabisch S. Pfeiffer A. E. H. Weickert M. O. (2020). The health benefits of dietary fibre. *Nutrients* 12:3209. 10.3390/nu12103209 33096647 PMC7589116

[B2] Beukema M. Faas M. M. de Vos P. (2020). The effects of different dietary fiber pectin structures on the gastrointestinal immune barrier: Impact via gut microbiota and direct effects on immune cells. *Exp. Mol. Med.* 52 1364–1376. 10.1038/s12276-020-0449-2 32908213 PMC8080816

[B3] Burokas A. Arboleya S. Moloney R. D. Peterson V. L. Murphy K. Clarke G. (2017). Targeting the microbiota-gut-brain axis: Prebiotics have anxiolytic and antidepressant-like effects and reverse the impact of chronic stress in mice. *Biol. Psychiatry* 82 472–487. 10.1016/j.biopsych.2016.12.031 28242013

[B4] Cox M. A. Jackson J. Stanton M. Rojas-Triana A. Bober L. Laverty M. (2009). Short-chain fatty acids act as antiinflammatory mediators by regulating prostaglandin E(2) and cytokines. *World J. Gastroenterol.* 15 5549–5557. 10.3748/wjg.15.5549 19938193 PMC2785057

[B5] Czirr E. Wyss-Coray T. (2012). The immunology of neurodegeneration. *J. Clin. Invest.* 122 1156–1163. 10.1172/JCI58656 22466657 PMC3315444

[B6] Erny D. Hrabe de Angelis A. L. Jaitin D. Wieghofer P. Staszewski O. David E. (2015). Host microbiota constantly control maturation and function of microglia in the CNS. *Nat. Neurosci.* 18 965–977. 10.1038/nn.4030 26030851 PMC5528863

[B7] Fernstrand A. M. Bury D. Garssen J. Verster J. C. (2017). Dietary intake of fibers: Differential effects in men and women on perceived general health and immune functioning. *Food Nutr. Res.* 61:1297053. 10.1080/16546628.2017.1297053 28469542 PMC5404421

[B8] Foster J. A. Neufeld K. A. M. (2013). Gut-brain: How the microbiome influences anxiety and depression. *Trends Neurosci.* 36 305–312. 10.1016/j.tins.2013.01.005 23384445

[B9] Guan Z. W. Yu E. Z. Feng Q. (2021). Soluble dietary fiber, one of the most important nutrients for the gut microbiota. *Molecules* 26:6802. 10.3390/molecules26226802 34833893 PMC8624670

[B10] Holscher H. D. (2017). Dietary fiber and prebiotics and the gastrointestinal microbiota. *Gut Microbes* 8 172–184. 10.1080/19490976.2017.1290756 28165863 PMC5390821

[B11] Jiang T. T. Gao X. J. Wu C. Tian F. Lei Q. C. Bi J. C. (2016). Apple-derived pectin modulates gut microbiota, improves gut barrier function, and attenuates metabolic endotoxemia in rats with diet-induced obesity. *Nutrients* 8:126. 10.3390/nu8030126 26938554 PMC4808856

[B12] Kellar K. L. Kalwar R. R. Dubois K. A. Crouse D. Chafin W. D. Kane B. E. (2001). Multiplexed fluorescent bead-based immunoassays for quantitation of human cytokines in serum and culture supernatants. *Cytometry* 45 27–36. 10.1002/1097-0320(20010901)45:1<27::AID-CYTO1141>3.0.CO;2-I 11598944

[B13] Lazic S. E. Essioux L. (2013). Improving basic and translational science by accounting for litter-to-litter variation in animal models. *BMC Neurosci.* 14:37. 10.1186/1471-2202-14-37 23522086 PMC3661356

[B14] Lazic S. E. Clarke-Williams C. J. Munafo M. R. (2018). What exactly is ‘N’ in cell culture and animal experiments? *PLoS Biol.* 16:e2005282. 10.1371/journal.pbio.2005282 29617358 PMC5902037

[B15] Macfarlane G. T. Macfarlane S. (2012). Bacteria, colonic fermentation, and gastrointestinal health. *J. AOAC Int.* 95 50–60. 10.5740/jaoacint.SGE_Macfarlane 22468341

[B16] Mailing L. J. Allen J. M. Pence B. D. Rytych J. Sun Y. Bhattacharya T. K. (2019). Behavioral response to fiber feedingis cohort-dependent and associated with gut microbiota composition in mice. *Behav. Brain Res.* 359 731–736. 10.1016/j.bbr.2018.09.012 30243767

[B17] Marrocco F. Delli Carpini M. Garofalo S. Giampaoli O. De Felice E. Di Castro M. A. (2022). Short-chain fatty acids promote the effect of environmental signals on the gut microbiome and metabolome in mice. *Commun. Biol.* 5:517. 10.1038/s42003-022-03468-9 35641653 PMC9156677

[B18] Matt S. M. Allen J. M. Lawson M. A. Mailing L. J. Woods J. A. Johnson R. W. (2018). Butyrate and dietary soluble fiber improve neuroinflammation associated with aging in mice. *Front. Immunol.* 9:1832. 10.3389/fimmu.2018.01832 30154787 PMC6102557

[B19] Merheb R. Abdel-Massih R. M. Karam M. C. (2019). Immunomodulatory effect of natural and modified Citrus pectin on cytokine levels in the spleen of BALB/c mice. *Int. J. Biol. Macromol.* 121 1–5. 10.1016/j.ijbiomac.2018.09.189 30292091

[B20] Miller A. H. Haroon E. Raison C. L. Felger J. C. (2013). Cytokine targets in the brain: Impact on neurotransmitters and neurocircuits. *Depress Anxiety* 30 297–306. 10.1002/da.22084 23468190 PMC4141874

[B21] Nicholson J. K. Holmes E. Kinross J. Burcelin R. Gibson G. Jia W. (2012). Host-gut microbiota metabolic interactions. *Science* 336 1262–1267. 10.1126/science.1223813 22674330

[B22] Paderin N. M. Popov S. V. (2018). The effect of dietary pectins on object recognition memory, depression-like behaviour, and IL-6 in mouse hippocampi. *J. Funct. Foods* 43 131–138. 10.1016/j.jff.2018.02.015

[B23] Patnala R. Arumugam T. V. Gupta N. Dheen S. T. (2017). HDAC inhibitor sodium butyrate-mediated epigenetic regulation enhances neuroprotective function of microglia during ischemic stroke. *Mol. Neurobiol.* 54 6391–6411. 10.1007/s12035-016-0149-z 27722928

[B24] Raison C. L. Capuron L. Miller A. H. (2006). Cytokines sing the blues: Inflammation and the pathogenesis of depression. *Trends Immunol.* 27 24–31. 10.1016/j.it.2005.11.006 16316783 PMC3392963

[B25] Ramin S. Mysz M. A. Meyer K. Capistrant B. Lazovich D. Prizment A. (2020). A prospective analysis of dietary fiber intake and mental health quality of life in the Iowa Women’s Health Study. *Maturitas* 131 1–7. 10.1016/j.maturitas.2019.10.007 31787141 PMC6916712

[B26] Reisenauer C. J. Bhatt D. P. Mitteness D. J. Slanczka E. R. Gienger H. M. Watt J. A. (2011). Acetate supplementation attenuates lipopolysaccharide-induced neuroinflammation. *J. Neurochem.* 117 264–274. 10.1111/j.1471-4159.2011.07198.x 21272004 PMC3070819

[B27] Romo-Araiza A. Gutierrez-Salmean G. Galvan E. J. Hernandez-Frausto M. Herrera-Lopez G. Romo-Parra H. (2018). Probiotics and prebiotics as a therapeutic strategy to improve memory in a model of middle-aged rats. *Front. Aging Neurosci.* 10:416. 10.3389/fnagi.2018.00416 30618722 PMC6305305

[B28] Rosen G. D. Williams A. G. Capra J. A. Connolly M. T. Cruz B. Lu L. (2000). *The mouse brain library.* Available online at: http://www.mbl.org (accessed September 9, 2021).

[B29] Saghafian F. Hajishafiee M. Rouhani P. Saneei P. (2022). Dietary fiber intake, depression, and anxiety: A systematic review and meta-analysis of epidemiologic studies. *Nutr. Neurosci.* 26 108–126. 10.1080/1028415X.2021.2020403 36692989

[B30] Sahasrabudhe N. M. Beukema M. Tian L. M. Troost B. Scholte J. Bruininx E. (2018). Dietary fiber pectin directly blocks toll-like receptor 2-1 and prevents doxorubicin-induced ileitis. *Front. Immunol.* 9:383. 10.3389/fimmu.2018.00383 29545800 PMC5839092

[B31] Shi H. Ge X. Ma X. Zheng M. Cui X. Pan W. (2021). A fiber-deprived diet causes cognitive impairment and hippocampal microglia-mediated synaptic loss through the gut microbiota and metabolites. *Microbiome* 9:223. 10.1186/s40168-021-01172-0 34758889 PMC8582174

[B32] Silva Y. P. Bernardi A. Frozza R. L. (2020). The role of short-chain fatty acids from gut microbiota in gut-brain communication. *Front. Endocrinol. (Lausanne)* 11:25. 10.3389/fendo.2020.00025 32082260 PMC7005631

[B33] Singh V. Yeoh B. Vijay-Kumar M. (2020). Fiber pectin improves intestinal inflammation by modulating gut microbial metabolites and inflammasome activity. *Curr. Dev. Nutr.* 29 (4 Suppl. 2) 1535. 10.1093/cdn/nzaa068_020

[B34] Smith J. G. Yokoyama W. H. German J. B. (1998). Butyric acid from the diet: Actions at the level of gene expression. *Crit. Rev. Food Sci. Nutr.* 38 259–297. 10.1080/10408699891274200 9626487

[B35] Soliman M. L. Smith M. D. Houdek H. M. Rosenberger T. A. (2012). Acetate supplementation modulates brain histone acetylation and decreases interleukin-1beta expression in a rat model of neuroinflammation. *J. Neuroinflammation* 9:51. 10.1186/1742-2094-9-51 22413888 PMC3317831

[B36] Stellwagen D. Malenka R. C. (2006). Synaptic scaling mediated by glial TNF-alpha. *Nature* 440 1054–1059. 10.1038/nature04671 16547515

[B37] Sun Y. J. He Y. Wang F. Zhang H. de Vos P. Sun J. (2017). Low-methoxyl lemon pectin attenuates inflammatory responses and improves intestinal barrier integrity in caerulein-induced experimental acute pancreatitis. *Mol. Nutr. Food Res.* 61:1600885. 10.1002/mnfr.201600885 27921358

[B38] Szklany K. Wopereis H. de Waard C. van Wageningen T. An R. van Limpt K. (2020). Supplementation of dietary non-digestible oligosaccharides from birth onwards improve social and reduce anxiety-like behaviour in male BALB/c mice. *Nutr. Neurosci.* 23 896–910. 10.1080/1028415X.2019.1576362 30871432

[B39] Tian L. Scholte J. Borewicz K. van den Bogert B. Smidt H. Scheurink A. J. (2016). Effects of pectin supplementation on the fermentation patterns of different structural carbohydrates in rats. *Mol. Nutr. Food Res.* 60 2256–2266. 10.1002/mnfr.201600149 27174558

[B40] van de Wouw M. Boehme M. Lyte J. M. Wiley N. Strain C. O’Sullivan O. (2018). Short-chain fatty acids: Microbial metabolites that alleviate stress-induced brain-gut axis alterations. *J. Physiol.* 596 4923–4944. 10.1113/JP276431 30066368 PMC6187046

[B41] Vazquez E. Barranco A. Ramirez M. Gruart A. Delgado-Garcia J. M. Martinez-Lara E. (2015). Effects of a human milk oligosaccharide, 2’-fucosyllactose, on hippocampal long-term potentiation and learning capabilities in rodents. *J. Nutr. Biochem.* 26 455–465. 10.1016/j.jnutbio.2014.11.016 25662731

[B42] Vignali D. A. (2000). Multiplexed particle-based flow cytometric assays. *J. Immunol. Methods* 243 243–255. 10.1016/S0022-1759(00)00238-6 10986418

[B43] Vogt L. M. Sahasrabudhe N. M. Ramasamy U. Meyer D. Pullens G. Faas M. M. (2016). The impact of lemon pectin characteristics on TLR activation and T84 intestinal epithelial cell barrier function. *J. Funct. Foods* 22 398–407. 10.1016/j.jff.2016.02.002

[B44] Voragen A. G. J. Coenen G. J. Verhoef R. P. Schols H. A. (2009). Pectin, a versatile polysaccharide present in plant cell walls. *Struct. Chem.* 20 263–275. 10.1007/s11224-009-9442-z

[B45] Wang P. Zhang Y. Gong Y. Yang R. Chen Z. Hu W. (2018). Sodium butyrate triggers a functional elongation of microglial process via Akt-small RhoGTPase activation and HDACs inhibition. *Neurobiol. Dis.* 111 12–25. 10.1016/j.nbd.2017.12.006 29248540

[B46] Wattick R. A. Hagedorn R. L. Olfert M. D. (2018). Relationship between diet and mental health in a young adult appalachian college population. *Nutrients* 10:957. 10.3390/nu10080957 30044399 PMC6115820

[B47] Wei Y. Melas P. A. Wegener G. Mathe A. A. Lavebratt C. (2014). Antidepressant-like effect of sodium butyrate is associated with an increase in TET1 and in 5-hydroxymethylation levels in the Bdnf gene. *Int. J. Neuropsychopharmacol.* 18:yu032. 10.1093/ijnp/pyu032 25618518 PMC4368891

[B48] Wilms E. Jonkers D. M. A. E. Savelkoul H. F. J. Elizalde M. Tischmann L. de Vos P. (2019). The impact of pectin supplementation on intestinal barrier function in healthy young adults and healthy elderly. *Nutrients* 11:1554. 10.3390/nu11071554 31324040 PMC6683049

[B49] Yamawaki Y. Yoshioka N. Nozaki K. Ito H. Oda K. Harada K. (2018). Sodium butyrate abolishes lipopolysaccharide-induced depression-like behaviors and hippocampal microglial activation in mice. *Brain Res.* 1680 13–38. 10.1016/j.brainres.2017.12.004 29229502

[B50] Yirmiya R. Goshen I. (2011). Immune modulation of learning, memory, neural plasticity and neurogenesis. *Brain Behav. Immun.* 25 181–213. 10.1016/j.bbi.2010.10.015 20970492

[B51] Zhang S. Wang H. Zhu M. J. (2019). A sensitive GC/MS detection method for analyzing microbial metabolites short chain fatty acids in fecal and serum samples. *Talanta* 196 249–254. 10.1016/j.talanta.2018.12.049 30683360

