# Supplementary material for: Serum short chain fatty acids mediate hippocampal BDNF and correlate with decreasing neuroinflammation following high pectin fiber diet in mice
Source: Front Neurosci. 2023 Apr 11;17:1134080. doi: 10.3389/fnins.2023.1134080 (PMC10130583; doi:10.3389/fnins.2023.1134080)
Supplement: Supplementary file 1 [file Data_Sheet_1.docx]

**Supplementary Materials**

**Serum short chain fatty acids mediate hippocampal BDNF and correlate with decreasing neuroinflammation following high pectin fiber diet in mice**

Jamie S. Church^1^, Jane A.M. Bannish^1^, Leighelle A. Adrian^1^, Kimberly Rojas Martinez^1^, Asari Henshaw^1^, and Jared J. Schwartzer^1^*

| Table S1: Acetic | | | | | | | | | | | | | |
| --- | --- | --- | --- | --- | --- | --- | --- | --- | --- | --- | --- | --- | --- |
| Model Components | |  | Random-Effects Model | | |  | **Mixed-Effects Model 1**  **(Main Effects)** | | |  | Mixed-Effects Model 2  (Interactions) | | |
| Fixed effects | |  | β | SE | t |  | β | SE | t |  | β | SE | t |
|  | (Intercept) |  | 138 | 16 | 8.7* |  | 93 | 13 | 7.1* |  | 104.07 | 16 | 6.55* |
|  | Diet (Pectin) |  |  |  |  |  | 72 | 15 | 4.9* |  | 54.81 | 21 | 2.66 |
|  | Sex (Male) |  |  |  |  |  | 17 | 13 | 1.3 |  | -0.84 | 18 | -0.046 |
|  | Diet*Sex |  |  |  |  |  |  |  |  |  | 32.66 | 25 | 1.31 |
| Random effects | |  | σ^2^ | | |  |  | σ^2^ |  |  |  | σ^2^ |  |
|  | Litter |  | 1242.02 | | |  | 86.38 | | |  | 123.62 | | |
|  | Residual |  | 1662.96 | | |  | 1570.51 | | |  | 1479.54 | | |
| Estimate of Model Fit | |  | AIC | 𝜒^2^ | *p* |  | AIC | 𝜒^2^ | *p* |  | AIC | 𝜒^2^ | *p* |
|  | |  | 468.37 |  |  |  | 460.70 | 11.67 | 0.0029 |  | 460.91 | 1.79 | 0.18 |
| Table S2: Propionic | | | | | | | | | | | | | |
| Model Components | |  | **Random-Effects Model** | | |  | Mixed-Effects Model 1  (Main Effects) | | |  | Mixed-Effects Model 2  (Interactions) | | |
| Fixed effects | |  | β | SE | t |  | β | SE | t |  | β | SE | t |
|  | (Intercept) |  | 8.31 | 1.94 | 4.29* |  | 5.29 | 3.1 | 1.71 |  | 2.9 | 3.7 | 0.78 |
|  | Diet (Pectin) |  |  |  |  |  | 4.83 | 3.5 | 1.37 |  | 8.7 | 4.7 | 1.85 |
|  | Sex (Male) |  |  |  |  |  | 0.96 | 3 | 0.32 |  | 4.9 | 4.3 | 1.13 |
|  | Diet*Sex |  |  |  |  |  |  |  |  |  | -7.3 | 5.9 | -1.24 |
| Random effects | |  | σ^2^ | | |  |  | σ^2^ |  |  |  | σ^2^ |  |
|  | Litter |  | 10.169 | | |  | 5.08 | | |  | 5.12 | | |
|  | Residual |  | 86.52 | | |  | 86.04 | | |  | 82.72 | | |
| Estimate of Model Fit | |  | AIC | 𝜒^2^ | *p* |  | AIC | 𝜒^2^ | *p* |  | AIC | 𝜒^2^ | *p* |
|  | |  | 330.84 |  |  |  | 333.04 | 1.81 | 0.41 |  | 333.39 | 1.65 | 0.20 |
| Table S3: Butyric | | | | | | | | | | | | | |
| Model Components | |  | **Random-Effects Model** | | |  | Mixed-Effects Model 1  (Main Effects) | | |  | Mixed-Effects Model 2  (Interactions) | | |
| Fixed effects | |  | β | SE | t |  | β | SE | t |  | β | SE | t |
|  | (Intercept) |  | 1.2 | 0.1 | 12* |  | 1.3058 | 0.19 | 6.99* |  | 1.1649 | 0.22 | 5.33* |
|  | Diet (Pectin) |  |  |  |  |  | 0.0054 | 0.21 | 0.025 |  | 0.2305 | 0.28 | 0.82 |
|  | Sex (Male) |  |  |  |  |  | -0.2313 | 0.18 | -1.30 |  | -0.0052 | 0.26 | -0.02 |
|  | Diet*Sex |  |  |  |  |  |  |  |  |  | -0.4211 | 0.36 | -1.18 |
| Random effects | |  | σ^2^ | | |  |  | σ^2^ |  |  |  | σ^2^ |  |
|  | Litter |  | 0.014 | | |  | 0.019 | | |  | 0.016 | | |
|  | Residual |  | 0.33 | | |  | 0.31 | | |  | 0.30 | | |
| Estimate of Model Fit | |  | AIC | 𝜒^2^ | *p* |  | AIC | 𝜒^2^ | *p* |  | AIC | 𝜒^2^ | *p* |
|  | |  | 83.35 |  |  |  | 85.54 | 1.82 | 0.4 |  | 86.03 | 1.51 | 0.22 |

| Table S: | | | | | | | | | | | | | |
| --- | --- | --- | --- | --- | --- | --- | --- | --- | --- | --- | --- | --- | --- |
| Model Components | |  | Random-Effects Model | | |  | **Mixed-Effects Model 1**  **(Main Effects)** | | |  | Mixed-Effects Model 2  (Interactions) | | |
| Fixed effects | |  | β | SE | t |  | β | SE | t |  | β | SE | t |
|  | (Intercept) |  | 3.6 | 0.32 | 11* |  | 4.87 | 0.72 | 6.78* |  | 4.98 | 0.69 | 7.2* |
|  | Diet (Pectin) |  |  |  |  |  | -2.8 | 0.64 | -4.34* |  | -2.95 | 0.67 | -4.4* |
|  | Sex (Male) |  |  |  |  |  | 0.026 | 0.33 | 0.079 |  | -0.38 | 0.48 | -0.8 |
|  | Diet*Sex |  |  |  |  |  |  |  |  |  | 0.75 | 0.66 | 1.1 |
| Random effects | |  | σ^2^ | | |  |  | σ^2^ |  |  |  | σ^2^ |  |
|  | Litter |  | 0.23 | | |  | 1.44 | | |  | 1.19 | | |
|  | Residual |  | 1.35 | | |  | 0.72 | | |  | 0.71 | | |
| Estimate of Model Fit | |  | AIC | 𝜒^2^ | *p* |  | AIC | 𝜒^2^ | *p* |  | AIC | 𝜒^2^ | *p* |
|  | |  | 109.71 |  |  |  | 101.71 | 12.01 | 0.0025 |  | 102.28 | 1.42 | 0.23 |
| Table S5: IL-1β | | | | | | | | | | | | | |
| Model Components | |  | Random-Effects Model | | |  | **Mixed-Effects Model 1**  **(Main Effects)** | | |  | Mixed-Effects Model 2  (Interactions) | | |
| Fixed effects | |  | β | SE | t |  | β | SE | t |  | β | SE | t |
|  | (Intercept) |  | 4.2 | 0.44 | 9.6* |  | 4.99 | 0.58 | 8.65* |  | 4.96 | 0.61 | 8.12* |
|  | Diet (Pectin) |  |  |  |  |  | -1.13 | 0.65 | -1.73^+^ |  | -1.08 | 0.73 | -1.5 |
|  | Sex (Male) |  |  |  |  |  | -0.46 | 0.38 | -1.21 |  | -0.38 | 0.56 | -0.68 |
|  | Diet*Sex |  |  |  |  |  |  |  |  |  | -0.15 | 0.77 | -0.19 |
| Random effects | |  | σ^2^ | | |  |  | σ^2^ |  |  |  | σ^2^ |  |
|  | Litter |  | 0.60 | | |  | 0.68 | | |  | 0.69 | | |
|  | Residual |  | 1.21 | | |  | 0.99 | | |  | 0.99 | | |
| Estimate of Model Fit | |  | AIC | 𝜒^2^ | *p* |  | AIC | 𝜒^2^ | *p* |  | AIC | 𝜒^2^ | *p* |
|  | |  | 109.30 |  |  |  | 108.16 | 5.15 | 0.076 |  | 110.12 | 0.041 | 0.84 |
| Table S6: IL-6 | | | | | | | | | | | | | |
| Model Components | |  | Random-Effects Model | | |  | **Mixed-Effects Model 1**  **(Main Effects)** | | |  | Mixed-Effects Model 2  (Interactions) | | |
| Fixed effects | |  | β | SE | t |  | β | SE | t |  | β | SE | t |
|  | (Intercept) |  | 2.4 | 0.23 | 10 |  | 2.96 | 0.35 | 8.54* |  | 3.05 | 0.36 | 8.43* |
|  | Diet (Pectin) |  |  |  |  |  | -1.07 | 0.41 | -2.58* |  | -1.22 | 0.46 | -2.64* |
|  | Sex (Male) |  |  |  |  |  | -0.12 | 0.26 | -0.47 |  | -0.35 | 0.38 | -0.92 |
|  | Diet*Sex |  |  |  |  |  |  |  |  |  | 0.43 | 0.53 | 0.82 |
| Random effects | |  | σ^2^ | | |  |  | σ^2^ |  |  |  | σ^2^ |  |
|  | Litter |  | 0.1323848 | | |  | 0.1992732 | | |  | 0.1795789 | | |
|  | Residual |  | 0.6228071 | | |  | 0.4744864 | | |  | 0.4673866 | | |
| Estimate of Model Fit | |  | AIC | 𝜒^2^ | *p* |  | AIC | 𝜒^2^ | *p* |  | AIC | 𝜒^2^ | *p* |
|  | |  | 85.61 |  |  |  | 82.81 | 6.80 | 0.033 |  | 84.06 | 0.75 | 0.39 |

| Table S7: TNF 𝛼 | | | | | | | | | | | | | |
| --- | --- | --- | --- | --- | --- | --- | --- | --- | --- | --- | --- | --- | --- |
| Model Components | |  | Random-Effects Model | | |  | **Mixed-Effects Model 1**  **(Main Effects)** | | |  | Mixed-Effects Model 2  (Interactions) | | |
| Fixed effects | |  | β | SE | t |  | β | SE | t |  | β | SE | t |
|  | (Intercept) |  | 4.3 | 0.27 | 16* |  | 5.19 | 0.48 | 10.9* |  | 5.32 | 0.46 | 11.6* |
|  | Diet (Pectin) |  |  |  |  |  | -1.6 | 0.52 | -3.1* |  | -1.81 | 0.54 | -3.4 |
|  | Sex (Male) |  |  |  |  |  | -0.29 | 0.29 | -1 |  | -0.72 | 0.41 | -1.7 |
|  | Diet*Sex |  |  |  |  |  |  |  |  |  | 0.8 | 0.57 | 1.4 |
| Random effects | |  | σ^2^ | | |  |  | σ^2^ |  |  |  | σ^2^ |  |
|  | Litter |  | 0.18 | | |  | 0.51 | | |  | 0.40 | | |
|  | Residual |  | 0.87 | | |  | 0.56 | | |  | 0.54 | | |
| Estimate of Model Fit | |  | AIC | 𝜒^2^ | *p* |  | AIC | 𝜒^2^ | *p* |  | AIC | 𝜒^2^ | *p* |
|  | |  | 96.18 |  |  |  | 90.85 | 9.33 | 0.0094 |  | 90.69 | 2.16 | 0.14 |
| Table S8: BDNF | | | | | | | | | | | | | |
| Model Components | |  | Random-Effects Model | | |  | **Mixed-Effects Model 1**  **(Main Effects)** | | |  | Mixed-Effects Model 2  (Interactions) | | |
| Fixed effects | |  | β | SE | t |  | β | SE | t |  | β | SE | t |
|  | (Intercept) |  | 18 | 1.5 | 12* |  | 17.1 | 2.4 | 7.1* |  | 14.88 | 2.7 | 5.47* |
|  | Diet (Pectin) |  |  |  |  |  | 6.5 | 2.8 | 2.3* |  | 10.98 | 3.8 | 2.85* |
|  | Sex (Male) |  |  |  |  |  | -4.6 | 2.8 | -1.6 |  | -0.06 | 3.8 | -0.016 |
|  | Diet*Sex |  |  |  |  |  |  |  |  |  | -9.06 | 5.4 | -1.67 |
| Random effects | |  | σ^2^ | | |  |  | σ^2^ |  |  |  | σ^2^ |  |
|  | Litter |  | <0.001 | | |  | <0.001 | | |  | <0.001 | | |
|  | Residual |  | 7.27 | | |  | 5.70 | | |  | 5.19 | | |
| Estimate of Model Fit | |  | AIC | 𝜒^2^ | *p* |  | AIC | 𝜒^2^ | *p* |  | AIC | 𝜒^2^ | *p* |
|  | |  | 233.959 |  |  |  | 230.18 | 7.779 | 0.02 |  | 229.16 | 3.02 | 0.082 |
