# Supplementary figures and images for: Serum short chain fatty acids mediate hippocampal BDNF and correlate with decreasing neuroinflammation following high pectin fiber diet in mice
Source: Front Neurosci. 2023 Apr 11;17:1134080. doi: 10.3389/fnins.2023.1134080 (PMC10130583; doi:10.3389/fnins.2023.1134080)

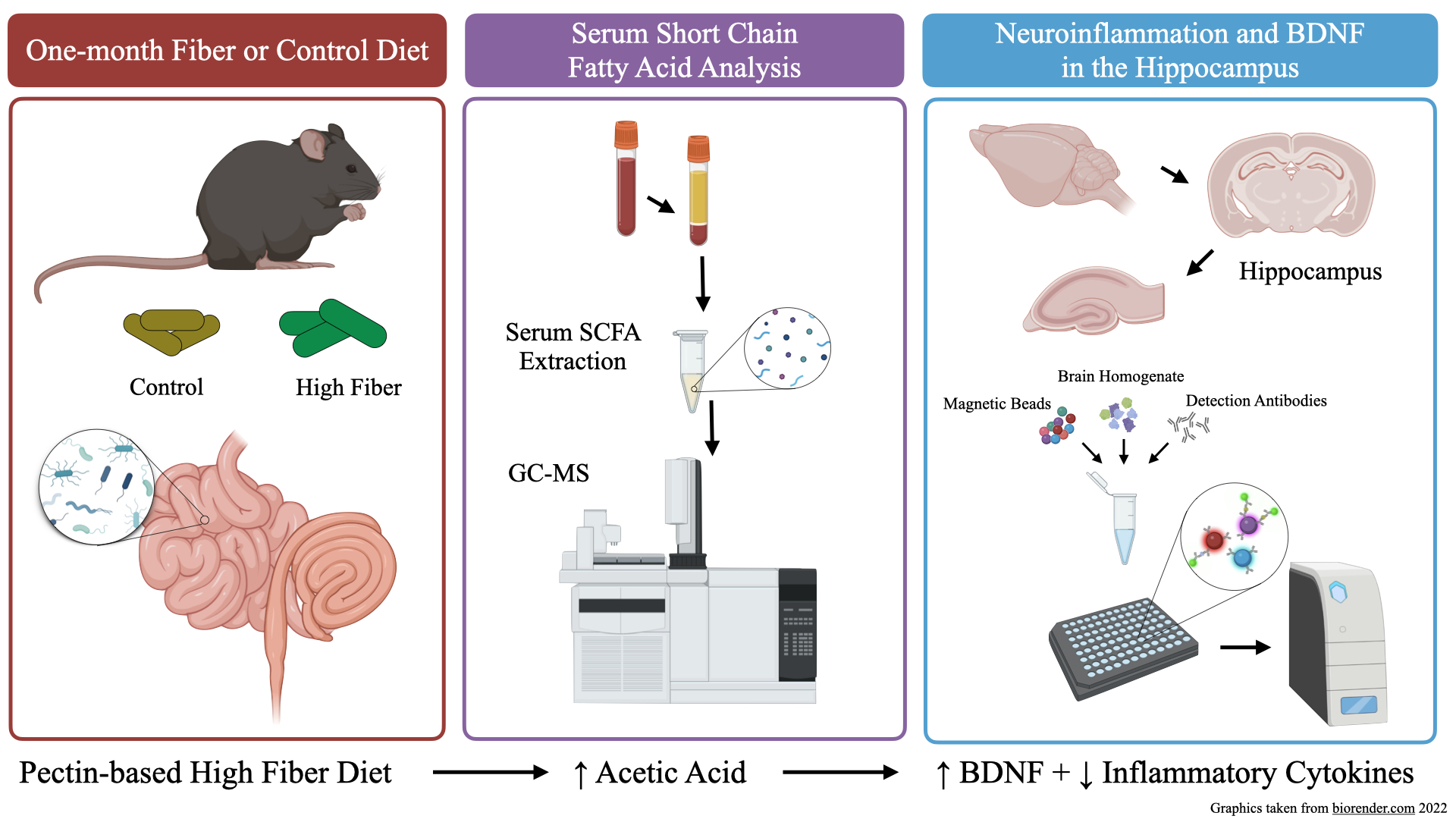

Supplement: Supplementary file 2 [file Image_1.JPEG]
